# Effect of Workplace Counseling Interventions Launched by Workplace Health Promotion and Tobacco Control Centers in Taiwan: An Evaluation Based on the Ottawa Charter

**DOI:** 10.1371/journal.pone.0150710

**Published:** 2016-03-08

**Authors:** Tzu-Hua Chen, Joh-Jong Huang, Fong-Ching Chang, Yu-Tsz Chang, Hung-Yi Chuang

**Affiliations:** 1 Department of Family Medicine, Kaohsiung Municipal Ta-Tung Hospital, Kaohsiung, Taiwan; 2 Department of Family Medicine, Kaohsiung Medical University Hospital, Kaohsiung, Taiwan; 3 Department of Health Promotion and Health Education, National Taiwan Normal University, Taipei, Taiwan; 4 Department of Public Health, College of Health Sciences, Kaohsiung Medical University, Kaohsiung, Taiwan; 5 Department of Occupational and Environmental Medicine, Kaohsiung Medical University Hospital, Kaohsiung, Taiwan; University of Illinois at Chicago, UNITED STATES

## Abstract

Workplace health promotion (WHP) is important to prevent work-related diseases, reduce workplace hazards, and improve personal health of the workers. Health promotion projects were launched through the centers of WHP funded by the Taiwan Bureau of Health Promotion since 2003. Hence, the aim of this study is to evaluate the impact of WHP programs intervention from 2003 to 2007. The intervention group consisted of 838 business entities which had ever undergone counseling of the three centers in northern, central, and southern Taiwan from 2003 to 2007. The control group was composed of 1000 business entities randomly selected from the business directories of the Ministry of Economic Affairs, Taiwan. The questionnaire survey included general company profiles and the assessment of workplace health according to the five action areas of the Ottawa Charter for Health Promotion. We have received 447 (53.3%) questionnaires from the intervention group and 97 questionnaires from the control group. The intervention group was more effective in using the external resources and medical consultation, and they had better follow-up rates of the abnormal results of annual health examinations. Compared to the control group, the intervention group had a significantly decreased smoking rate in 246 companies (61.2%) and a reduced second-hand smoke exposure in 323 companies (78.6%) (p<0.001). By means of the intervention of WHP programs, we can enhance the awareness of the enterprises and the employees to improve worksite health and to create a healthy work environment.

## Introduction

Health promotion was defined as “the process of enabling people to increase control over and improve their health” according to the Ottawa Charter for Health Promotion [1] issued by the World Health Organization (WHO) in 1986. When the idea of health promotion was introduced in 1970, many developed countries raised concerns over employees’ health conditions. In the 1980s, workplace health promotion (WHP) became the focus of wellness programs. WHP courses and activities were held to improve the workers’ physical condition, prevent chronic diseases, and reduce musculoskeletal disorders [2, 3]. Most workers spend one-third or more of the day at work, making the workplace the best platform for carrying out health promotion activities. Such activities not only improve employees’ physical health and reduce their sick leave rates [4], but can also enhance employees’ productivity and the corporate image. As a result, strengthening WHP is highly important in preventing work-related diseases, reducing occupational injuries, and improving workers’ health conditions.

With the transition of dietary habits and lifestyles, the disease types of the workers also changed. An increased digestion of western diet and great psychosocial stressors may predispose the workers to chronic diseases, such as cardiovascular diseases and musculoskeletal disorders [5–7]. To accommodate the demand for occupational hygiene and occupational health, Taiwan authorities gradually introduced the idea of WHP since 1990. The Bureau of Health Promotion, Department of Health funded several large-scale medical organizations and academic institutions to establish Workplace Hygiene and Healthcare Centers in 2001 to encourage WHP campaigns. Worksite Tobacco Control Consulting Centers were set up from 2003 to 2005 to promote smoke-free workplace. Moreover, in order to coordinate the business of WHP and tobacco control, the two centers have been integrated in 2006 to form Workplace Health Promotion and Tobacco Control Centers [8]. The three centers located in the northern, central, and southern regions of Taiwan enabled the enterprises to carry out WHP more thoroughly.

The advantages of WHP activities in other countries included work-related stress relief, sickness absence rate reduction, and improvement of workers’ health status [9]. There has been little information about the impact of WHP which was funded by the official organization in Taiwan. Based on the five action areas of the Ottawa Charter for Health Promotion, we devised this study to evaluate the impact of counseling intervention provided by the Workplace Health Promotion and Tobacco Control Centers in Taiwan.

## Materials and Methods

### Study population and intervention

The study protocol was approved by the Institutional Review Board (IRB) in Kaohsiung Medical University Hospital, and we followed the guidelines of the IRB of Kaohsiung Medical University Hospital for experimentation with human subjects. The intervention group consisted of 866 companies which underwent counseling from the Workplace Health Promotion and Tobacco Control Centers from 2003 to 2007. However, 28 subsidiary companies were incorporated into the parent companies for centralization of management of health promotion campaigns, and thus there were 838 companies enrolled finally. Various activities were conducted at the workplace in the intervention group, such as health education, diet education, physical fitness classes, and smoking cessation classes. In order to create the smoke-free workplace, smoking indoors was banned, and smoking areas were designated to reduce second-hand smoke exposure. The control group, which never underwent any counseling from 2003 to 2007, was randomly selected from the business directories of the Ministry of Economic Affairs, Taiwan, and 1000 companies were recruited.

### Study design

We utilized mail questionnaire survey to conduct this cross-sectional study. The framework of the questionnaire was based on the five action areas of the Ottawa Charter for Health Promotion, 1.building public health policy, 2.creating supportive environments, 3.strengthening workplace health activities, 4.developing personal skills, 5.re-orientating health care services toward prevention of illness and promotion of health [1]. Five scholars of WHP were invited for questionnaire validation in the expert meeting on March 31, 2007. Additional ten companies which had ever undergone counseling of the centers from 2003 to 2007 were selected to pretest questionnaire to check the validity, and to provide opinions for questionnaire modification. The questionnaire content was divided into two parts, including general company profiles and assessments of workplace health.

The reliability of the research instrument was verified by test-retest reliability. Those ten companies mentioned above received retests after 11 days, and p-values of the two matching results all exceeded 0.125, indicating no significant differences between results in pre-tests and re-tests.

### Statistical analysis

We analyzed the data with IBM SPSS version 19.0 (IBM inc., Armonk, NY, USA). Chi-square test and logistic regression were used for statistical analysis, and the alpha level was set at 0.05.

## Results

Among the 838 companies of the intervention group, 447 companies replied the questionnaire, and the response rate was 53.3%. On the other hand, among the 1000 companies of the control group, 97 companies made a reply, and the response rate was 9.7%. The questionnaire collection period was from August 20, 2008 to December 1, 2008.

During the period from 2003 to 2007, the completed questionnaire copies from the intervention group in 2007 totaled 146 (70.0%); the response rate was higher than any other counseling years: 2003 (51.1%), 2004 (42.8%), 2005 (52.3%), and 2006 (46.2%). Chi-square test showed a significant difference between the response rate and the counseling year (p = 0.041). (Table 1) Among the three major geographical regions in Taiwan, the companies which underwent the counseling of the southern center had the highest response rate of 65.6%. Chi-square test revealed a significant difference between the response rate and the region of centers (p = 0.039). (Table 1)

**Table 1:** Response rates of business entities which received counseling from 2003 to 2007.

Variable		Number of questionnaires		*p* ^a^
		Total	Returned (%)	
Business entities which received counseling in the three regions		838	447(53.3%)	
Year of counseling				0.041
	2003	137	70(51.1%)	
	2004	166	71(42.8%)	
	2005	155	81(52.3%)	
	2006	171	79(46.2%)	
	2007	209	146(70.0%)	
Regional centers				0.039
	North	292	148(50.7%)	
	Central	302	139(46.0%)	
	South	244	160(65.6%)	
Company scale				
	2006			0.369
	Large-scale enterprise	39	19(48.7%)	
	Medium-scale enterprise	44	14(31.8%)	
	Small-scale enterprise	88	46(52.3%)	
	2007			0.645
	Large-scale enterprise	77	47(61.0%)	
	Medium-scale enterprise	29	24(82.8%)	
	Small-scale enterprise	103	72(69.9%)	

^a^ Analyzed by Chi-square test.

With regard to company scale, the worksites of WHP did not establish it as a variable from 2003 to 2005; hence, only the companies which received counseling in 2006 and 2007 were analyzed. Among the business entities that received counseling in 2006, the questionnaire response rates of large-, medium- and small-scale enterprises were 48.7%, 31.8%, and 52.3%, respectively. The response rates in 2007 were 61.0%, 82.8%, and 69.9%, respectively. Chi-square test showed no significant difference between the response rate and the company scale. (Table 1)

### Basic company profiles of intervention group and control group

Table 2 shows the basic company profiles of the intervention group and the control group. Both groups are mainly composed of non-foreign companies, totaling 390 (88.2%) in the intervention group and 94 (96.9%) in the control group. About half (50.8%) of the companies in the intervention group assigned the responsibility of questionnaire completion to the department of environmental health and safety. In the control group, only 6.6% of the business entities assigned it to the department of environmental health and safety. Most of the business entities (41.7%) in the control group delegated the responsibility to their accounting department. A significant difference was seen between these two groups (p<0.001).

**Table 2:** Basic company profile of the intervention group and control group.

Variable		Category		Total	*p*^a^
		Control group	Intervention group		
Foreign company					0.1
	Non-foreign	94 (96.9%)	390 (88.2%)	484 (89.8%)	
	American	1 (1.0%)	15 (3.4%)	16 (3.0%)	
	Japanese	0 (0%)	18 (4.1%)	18 (3.3%)	
	European	0 (0%)	7 (1.6%)	7 (1.3%)	
	Others	2 (2.1%)	12 (2.7%)	14 (2.6%)	
Department for answering the questionnaire					<0.001
	Labor safety/ Environmental safety/ Safety and hygiene	6 (6.6%)	227 (50.8%)	233 (43.3%)	
	General affairs	12 (13.2%)	54 (12.1%)	66 (12.3%)	
	Human resources	3 (3.3%)	31 (6.9%)	34 (6.3%)	
	Accounting	38 (41.7%)	12 (2.7%)	50 (9.3%)	
	Management	18 (19.8%)	60 (13.4%)	78 (14.5%)	
	Others	14 (15.4%)	63 (14.1%)	77 (14.3%)	
Shift work policy					<0.001
	Yes	20 (21.1%)	271 (61.5%)	291 (53.5%)	
	No	75 (78.9%)	170 (38.5%)	245 (45.0%)	
Factory doctors/ nurses					<0.001
	Doctors/Nurses	6 (6.5%)	165 (36.9%)	171 (32.3%)	
	Neither	86 (93.5%)	273 (61.1%)	359 (67.7%)	
Average age of employees in the company					
	Male employees				0.147
	25 or under	1 (1.0%)	2 (0.5%)	3 (0.6%)	
	26–35	29 (29.9%)	153 (35.1%)	182 (34.1%)	
	36–45	41 (42.3%)	207 (47.5%)	248 (46.5%)	
	46–55	21 (21.6%)	67 (15.3%)	88 (16.5%)	
	56 or above	3 (3.1%)	5 (1.1%)	8 (1.5%)	
	No male employees	2 (2.1%)	2 (0.5%)	4 (0.8%)	
	Female employees				0.064
	25 or under	0 (0.0%)	5 (1.1%)	5 (0.9%)	
	26–35	38 (39.2%)	225 (51.6%)	263 (49.3%)	
	36–45	41 (42.3%)	160 (36.7%)	201 (37.7%)	
	46–55	13 (13.4%)	39 (9.0%)	52 (9.8%)	
	56 or above	1 (1.0%)	1 (0.2%)	2 (0.4%)	
	No female employees	4 (4.1%)	6 (1.4%)	10 (1.9%)	

^a^ Analyzed by Chi-square test.

In the intervention group, 271 business entities (61.5%) had shift work policies. There were 75 business entities (78.9%) without shift work in the control group, exhibiting a considerable difference from the intervention group (p<0.001). The number of companies with factory doctors or nurses in the intervention group was 165 (36.9%) but only 6 (6.5%) in the control group, showing a statistically significant difference (p<0.001). The two age ranges of 26–35 and 36–45 constituted the largest proportions of both male and female employees in intervention and control groups, but there was no significant difference (p>0.05).

### Condition and effectiveness of popularizing WHP in intervention group and control group

As shown in Table 3, compared to the control group, the intervention group exhibited more prominent effectiveness in the use of external resources (71.1%) and medical resources (82.0%), and higher ratio of management of employees with abnormal physical checkup results (76.9%). The number of business entities with establishment of health indicators was 177 (43.3%) in the intervention group, but was only 5 (5.7%) in the control group. 169 business entities (40.8%) in the intervention group created budgets for WHP tasks, while a mere 6 (6.4%) in the control group did likewise, resulting in significant statistical difference (p<0.001). However, the indicator of manager support in the intervention group was lower than that in the control group.

**Table 3:** Health promotion indicators and workplace tobacco hazard improvement in the intervention group and control group.

Variable		Group		Total	*p*^a^
		Control group	Intervention group		
Facilitate manager engagement in health promotion issues					0.003
	Yes	81 (89.0%)	310 (75.2%)	391 (77.7%)	
	No	10 (11.0%)	102 (24.8%)	112 (22.3%)	
Facilitate manager engagement in tobacco hazard control issues					0.003
	Yes	83 (91.2%)	318 (77.2%)	401 (79.7%)	
	No	8 (8.8%)	94 (22.8%)	102 (20.3%)	
Popularize health promotion or tobacco hazard control work using external resources					<0.001
	Yes	16 (18.4%)	293 (71.1%)	309 (61.9%)	
	No	71 (81.6%)	119 (28.9%	190 (38.1%)	
Popularize health promotion or tobacco hazard control work using medical resources					<0.001
	Yes	32 (34.4%)	336 (82.0%)	368 (73.2%)	
	No	61 (65.6%)	74 (18.0%)	135 (26.8%)	
Establish health indicators for evaluating the effectiveness of health promotion or tobacco hazard control					<0.001
	Yes	5 (5.7%)	177 (43.3%)	182 (36.6%)	
	No	83 (94.3%)	232 (56.7%)	315 (63.4%)	
Include employee sick leave rate as marker of effectiveness of healthy workplace promotion					0.003
	Yes	19 (20.9%)	151 (36.5%)	170 (33.7%)	
	No	72 (79.1%)	263 (63.5%)	335 (66.3%)	
Include tracking and managing abnormal physical checkup results as marker of effectiveness of healthy workplace promotion					<0.001
	Yes	37 (40.7%)	317 (76.9%)	354 (70.4%)	
	No	54 (59.3%)	95 (23.1%)	149 (29.6%)	
Create budgets for health promotion or tobacco hazard control					<0.001
	Yes	6 (6.4%)	169 (40.8%)	175 (34.4%)	
	No	88 (93.6%)	245 (59.2%)	333 (65.6%)	
Whether smoke can be smelled in the workplace					<0.001
	Yes				
	Only in smoking area	20 (21.3%)	274 (66.3%)	294 (58.0%)	
	Both in smoking and non- smoking areas	20 (21.3%)	13 (3.2%)	33 (6.5%)	
	No	54 (57.4%)	126 (30.5%)	180 (35.5%)	
Decline of smoking rate					<0.001
	Yes	20 (21.1%)	246 (61.2%)	266 (53.5%)	
	No	59 (62.1%)	135 (33.6%)	194 (39.0%)	
	Non-smokers	16 (16.8%)	21 (5.2%)	37 (7.5%)	
Improvement in secondhand smoke exposure					<0.001
	Yes	45 (49.5%)	323 (78.6%)	368 (73.3%)	
	No	46 (50.5%)	88 (21.4%)	134 (26.7%)	

^a^ Analyzed by Chi-square test.

The number of workplaces using employees’ sick leave rates as an indicator of advocating WHP was 151 (36.5%) in the intervention group, and 19 (20.9%) in the control group, exhibiting a significant statistical difference. The number of workplaces reporting reduction in the company’s smoking rates totaled 246 (61.2%) in the intervention group, while only 20 (21.1%) companies in the control group reported such reduction. In regard to secondhand smoke, 323 business entities (78.6%) in the intervention group indicated improvement in secondhand smoke exposure, while the number was 45 (49.5%) in the control group.

### Logistic regression analysis

Table 4 and Table 5 show that the ratios of using external and medical resources and setting health indicators for promoting employee health were higher in the intervention group than those in the control group. Furthermore, the ratios of tracking and managing employees with abnormal physical checkup results, using employees’ sick leave rates as an indicator of health promotion, and improving the secondhand smoke condition in factories were higher in the intervention group than those in the control group.

**Table 4:** Logistic regression analysis of the counseling effects on the implementation of the five action areas for health promotion of the WHO’s Ottawa Charter, all adjusted for age. [OR = odds ratio, 95% CI = 95% confidence interval].

	Manager engagement in health promotion issues OR (95% CI)	Manager engagement in tobacco hazard control issues OR (95% CI)	Use of external resources OR (95% CI)	Use of medical resources OR (95% CI)	Establish health indicators OR (95% CI)
Received counseling	0.201* (0.083–0.487)	0.276* (0.113–0.671)	4.371* (2.224–8.592)	5.872* (3.084–11.180)	7.324* (2.689–19.946)
Responsible department (environmental health and safety vs. others)	1.500 (0.892–2.520)	1.205 (0.706–2.509)	1.568 (0.922–2.668)	0.917 (0.505–1.666)	1.706* (1.035–2.812)
Company nature (non-foreign vs. foreign)	0.836 (0.413–1.695)	0.685 (0.342–1.372)	2.100 (0.897–4.916)	0.983 (0.423–2.288)	0.638 (0.315–1.295)
Company scale					
Small vs. Large	1.751 (0.876–3.501)	1.683 (0.824–3.436)	0.384* (0.196–0.752)	0.629 (0.293–1.351)	1.852 (0.920–3.729)
Medium-small vs. Large	1.769 (0.931–3.359)	2.344* (1.177–4.669)	0.546 (0.289–1.029)	0.769 (0.375–1.581)	1.555 (0.839–2.881)
Medium vs. Large	1.727 (0.841–3.548)	1.708 (0.822–3.548)	0.928 (0.444–1.939)	1.353 (0.569–3.221)	1.599 (0.815–3.136)
With shift work policy	1.251 (0.749–2.088)	1.142 (0.669–1.951)	1.050 (0.638–1.729)	0.797 (0.455–1.396)	1.280 (0.773–2.117)
With factory doctors/ nurses	1.957* (1.081–3.541)	1.587 (0.872–2.888)	1.600 (0.870–2.943)	4.560* (2.136–9.732)	2.664* (1.542–4.60)

*p < 0.05.

**Table 5:** Logistic regression analysis of the counseling effects on the implementation of the five action areas for health promotion of the WHO’s Ottawa Charter, all adjusted for age. [OR = odds ratio, 95% CI = 95% confidence interval].

	Track and manage employees’ abnormal health checkup results OR(95% CI)	Include employees’ sick leave rate as indicator OR (95% CI)	Improvement of secondhand smoke at workplace OR (95% CI)	Smelling smoke only in the smoking area at workplace OR (95% CI)
Received counseling	2.556* (1.368–4.778)	3.591* (1.759–7.331)	2.383* (1.239–4.583)	1.797* (1.005–3.212)
Responsible department (environment health and safety vs. others)	1.102 (0.620–1.959)	1.566 (0.940–2.611)	0.854 (0.434–1.680)	1.637* (1.001–2.676)
Company nature (non-foreign vs. foreign)	0.454* (0.212–0.969)	0.793 (0.391–1.609)	2.056 (0.748–5.654)	0.763 (0.391–1.488)
Company scale				
Small vs. Large	0.506 (0.245–1.043)	3.557* (1.827–6.926)	0.044* (0.018–0.107)	0.648 (0.345–1.216)
Medium-small vs. Large	0.906 (0.447–1.836)	3.006* (1.662–5.439)	0.236* (0.106–0.527)	1.277 (0.692–2.356)
Medium vs. Large	1.509 (0.637–3.572)	1.579 (0.802–3.106)	0.183* (0.076–0.437)	0.978 (0.502–1.903)
With shift work policy	2.204* (1.298–3.740)	1.223 (0.751–1.991)	1.030 (0.558–1.901)	1.069 (0.662–1.727)
With factory doctors/ nurses	2.265* (1.132–4.534)	0.861 (0.491–1.511)	0.164* (0.074–0.362)	1.151 (0.663–2.000)

*p < 0.05.

Business entities with factory doctors or nurses had a higher percentage of manager engagement in health promotion issues, use of external and medical resources, and follow-up on employees with abnormal physical checkup results than those without factory doctors or nurses. In addition, business entities who assigned the department of environmental health and safety to be responsible for WHP affairs were 1.706 times more likely to set health indicators than if it was the responsibility of other departments.

Small-scale enterprises had the highest ratio for setting employees’ sick leave rates as an indicator, which was 3.557 times that of large-scale enterprises. The improvement of secondhand smoke situations in the workplaces of small-scale enterprises had the lowest ratio, and was only 0.044 times of that of large-scale enterprises, which indicated ineffective improvement of the secondhand smoke situation.

## Discussion

Following the five action areas of the Ottawa Charter for Health Promotion issued by WHO [1], many developed countries have incorporated these ideas in activities to popularize health promotion, such as smoking cessation and physical fitness classes. Research findings showed that the popularization of health promotion activities contributes to improvements in employees’ health conditions or their productivity [10, 11]. To familiarize business entities with the idea of WHP in Taiwan, the Bureau of Health Promotion, Department of Health has been advocating “smoke-free workplaces” since 2003 and “workplace health promotion and tobacco control” since 2005. The concepts of the five action areas for health promotion were incorporated into this study’s questionnaire to observe the centers’ effectiveness of counseling intervention for business entities. The investigation focused on the indicators of the condition and effectiveness of carrying out WHP.

### Building public health policy

Table 3 reveals that the intervention group experienced changes, such as smelling smoke only in the smoking area and improvement in secondhand smoke exposure. This indicated a more prominent decrease in the companies’ overall smoking rates than the control group. Due to the ban on smoking in indoor workplaces with three or more people according to the Tobacco Hazards Prevention Act, as well as the centers’ spreading of the smoke-free workplace idea, the smoking situation in companies of the intervention group improved as the workers had a deeper understanding of the importance of smoke-free policies. They also assisted in designating the smoking area at workplace. Other literature also mentioned that smoking ban implementation in business entities would reduce workplace smoking rates and secondhand smoke exposure [12–14].

### Creating supportive environments

The results show that the ratio of manager engagement in health promotion and tobacco hazard control in the control group was higher than in the intervention group. Prior literature indicated that managers played important roles in handling employees’ stress [15]. Manager support in the company is helpful to improving employees’ mental problems, such as depression and frustration [16, 17]. Moreover, managers can obtain relevant health information through education or training to help improve employees’ mental health [17, 18]. The results of this study reflect that the managers in the intervention group were less engaged in health promotion and tobacco hazard control than that in the control group. The reason may be that they could not participate in the activities in person due to busy meeting schedules or time conflicts. However, a higher ratio of business entities in the intervention group created budgets for health promotion and tobacco hazard control tasks than the control group, reflecting that the managers in the intervention group had considerable support for and concern over the issues. Hence, they created budgets specifically for health promotion and tobacco hazard control to improve employees’ physical and mental health conditions.

### Strengthening workplace health activities

Our analyses show that the ratios of tracking employees’ abnormal physical checkup results and managing their sick leave rates in the intervention group were higher than in the control group. Prior literature mentioned that employee healthcare following health checkups was one of the important aspects of workplace health, so that both the business entities and the employees understand the health conditions clearly. In addition, Eija Nurminen et al. found that advocating health promotion courses, such as physical fitness and smoking cessation classes, can lower employees’ sick leave rates and enhance productivity [11, 19]. Based on our results and literature review, business entities developed stronger motivation to manage employees’ health and to provide health promotion courses to enhance their health through the counseling provided by the three regional centers under the Bureau of Health Promotion.

### Developing personal skills

The intervention group had higher ratios of using external resources (e.g., community services), medical resources (e.g., Department of Health, Workplace Health Promotion and Tobacco Control Centers), and health indicators than the control group, illustrating a better understanding of the channels to obtain information and resources. We also observed indicators to examine whether the business entities guided their employees to find health promotion resources or to seek health information consultations in order to improve their health. Prior literature suggested encouraging the formation of contact networks between business entities and the community services. Thus, employees and residents could offer mutual help in improving health, and the health of small-scale business employees would not be neglected [20]. Furthermore, one study revealed that community health promotion courses provided appropriate outlets for work stress, offering a solution to employees’ mental health problems [21].

### Re-orientating health care services toward prevention of illness and promotion of health

Our results show more prominent improvement in the secondhand smoke problem in the intervention group than in the control group. Other literature also shared similar viewpoints. A research in Japan found that diverse health promotion or illness prevention courses for employees were effective in improving chronic diseases, such as hypertension, hyperlipidemia, and obesity [22]. Moreover, some researches on small- and medium- scale enterprises that organized hygiene education courses after annual health checkups revealed that the employees who attended four or more hygiene education courses had lower need for medical treatment for their chronic diseases. Hence, business entities can improve employees’ health conditions by providing them with health promotion or hygiene education courses [23].

### Strengths and limitations

This is the first nationwide study to evaluate the effectiveness of WHP programs which were funded by the Taiwan Bureau of Health Promotion. It is also the first questionnaire survey based on the five action areas of the Ottawa Charter for Health Promotion in Taiwan. However, this research was constrained by the locations of Workplace Health Promotion and Tobacco Control Centers. As this research was primarily conducted in the southern center, the business entities in northern and central Taiwan were not familiar with the questionnaire investigation. They were hesitant in answering the questionnaires, causing questionnaire collection in the north region (50.7%) and the central region (46.0%) to be less effective than in the south region (65.6%). Had the business entities in northern and central Taiwan been informed of the questionnaire evaluation at the beginning of this research, the response rate might have been increased, thereby increasing the centers’ effectiveness in counseling the business entities. In addition, the highest response rate was found to be the business entities counseled in 2007. The reason might be that the interval between counseling and questionnaire completion was less than one year. Hence, their impression of the counseling courses was deeper, and it was easier for them to complete the questionnaires.

## Conclusions

The aim of this research was to investigate the effectiveness of WHP under the counseling of Workplace Health Promotion and Tobacco Control Centers in three regions in Taiwan. By comparing the differences between the intervention group and the control group, the promotion of workplace health by three regional counseling centers was found to be effective in raising the business entities’ awareness, announcing the regulations, and improving employees’ health and working environment quality.
